# 

**Published:** 1909-06

**Authors:** 


					﻿Vol. LX1V.	JUNE, 1909.	No. u
BUFFALO
MEDICAL JOURNAL-.
V "	:  C ,nc,Oj<®ci
AUSTIN PLINT \^SP.G
JUN. 2-
' EDITOR:	*
WILLIAM WARREN POTTER, M. D.
ASSISTANT EDITORS:
WILLIAM C. KRAUoS, M. D.	NELSON W. WILSON, M. D
ASSOCIATE EDITORS:
JAMES WRIGHT PUTNAM, M. D. ERNEST WENDE, M.D.
JOHN PARMENTER, M. D.	JOHN A. MILLER, Ph. D.
‘	MAUD J. FRYE, M. D.
				

